# Supplementary material for: Poverty eradication in a carbon constrained world
Source: Nat Commun. 2017 Oct 24;8:912. doi: 10.1038/s41467-017-00919-4 (PMC5783954; doi:10.1038/s41467-017-00919-4)
Supplement: Supplementary file 1 — Supplementary Information [file 41467_2017_919_MOESM1_ESM.pdf]

Poverty eradication in a carbon constrained world

Klaus Hubacek, Giovanni Baiocchi, Kuishuang Feng, Anand Patwardhan

Supplementary figures

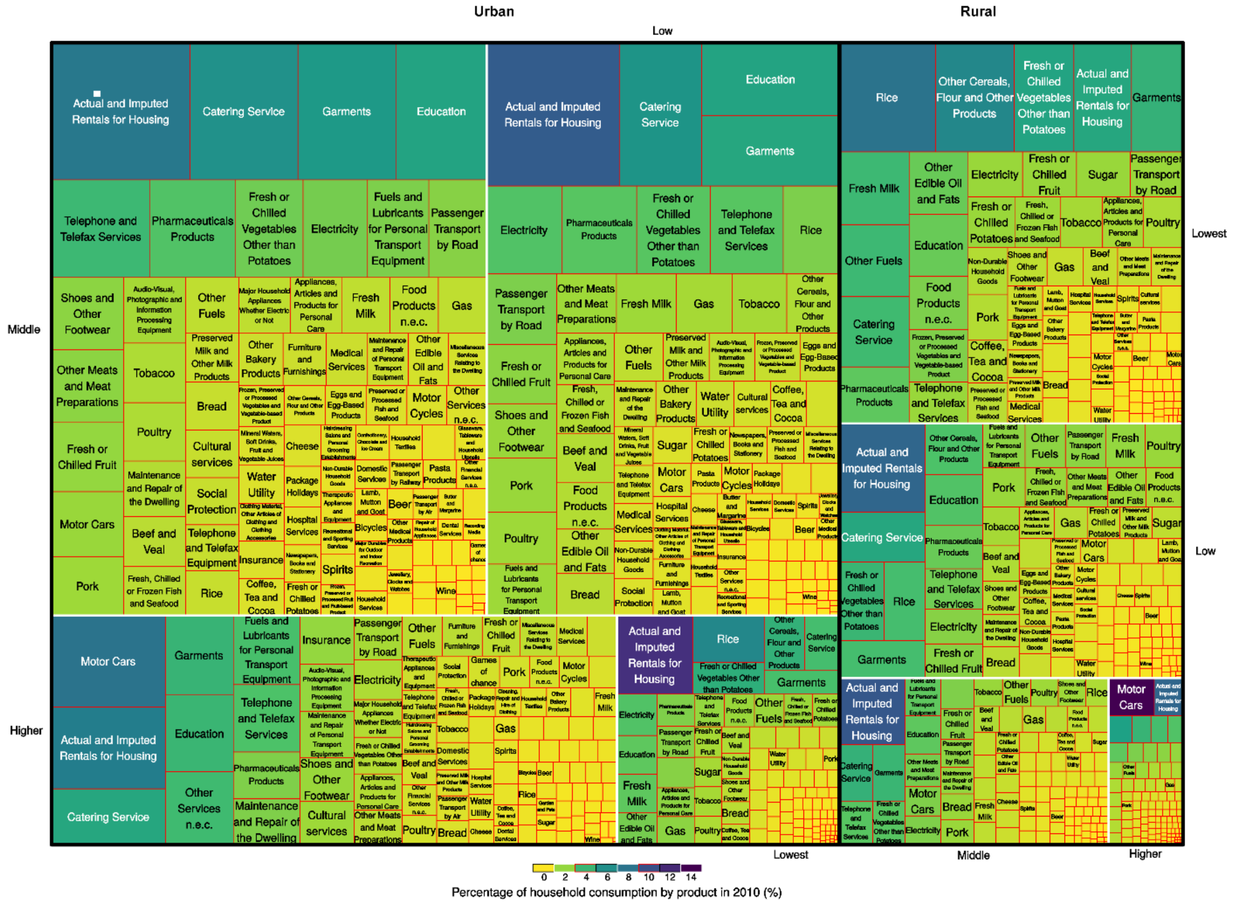

Supplementary Figure 1: Share of household consumption for different product and service categories by area and by income. Plot showing the percentage of household consumption for different product and service categories by area (rural and urban) and by income group (Lowest, Low, Middle, and Higher) for the 90 countries in the 2010 World Bank's Global Consumption Database. Area of the rectangles are proportional to total expenditures.

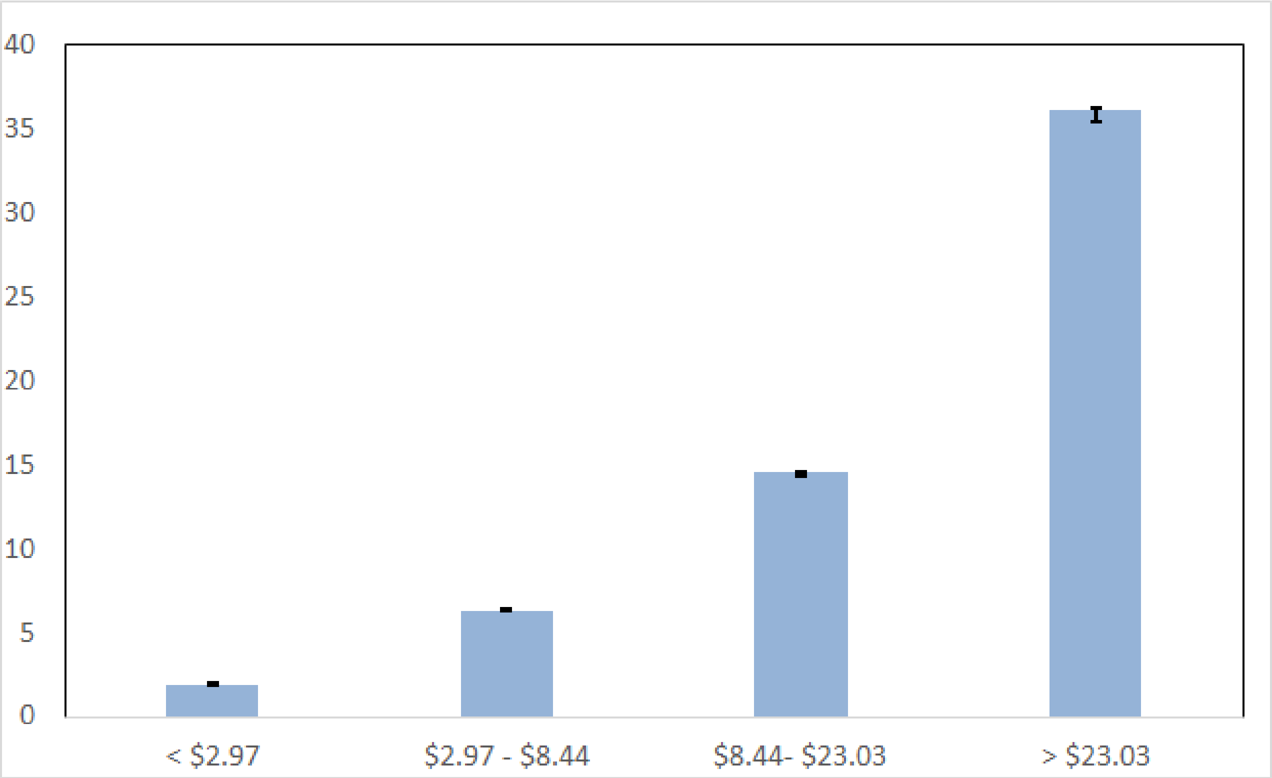

Supplementary Figure 2: Uncertainty analysis for per capita carbon footprint of four household groups

Supplementary tables

Supplementary Table 1: Average share and standard deviation of main consumption items for extreme poverty and low expenditure household groups

	Extreme Poverty		Low Expenditure	
	Average share of consumption	SD	Average share of consumption	SD
Food and Beverages	0.60	0.10	0.58	0.10
Clothing and Footwear	0.06	0.02	0.06	0.03
Housing	0.08	0.06	0.09	0.07

The table shows the average and standard deviation for three major expenditure categories showing that for the extremely poor (less than $1.9) and the less than $2.97 category the three consumption items account for about three quarters of their household budget across the 90 poorest countries.
