# Supplementary material for: Poverty eradication in a carbon constrained world
Source: Nat Commun. 2017 Oct 24;8:912. doi: 10.1038/s41467-017-00919-4 (PMC5783954; doi:10.1038/s41467-017-00919-4)
Supplement: Supplementary file 3 — Description of Additional Supplementary Files [file 41467_2017_919_MOESM3_ESM.pdf]

Description of Additional Supplementary Files

File Name: Supplementary Data 1
Description: Bridge Matrices
